# Should countries implementing an artemisinin-based combination malaria treatment policy also introduce rapid diagnostic tests?

**DOI:** 10.1186/1475-2875-7-176

**Published:** 2008-09-15

**Authors:** Charlotte M Zikusooka, Diane McIntyre, Karen I Barnes

**Affiliations:** 1 Health Economics Unit, Department of Public Health and Family Medicine, University of Cape Town, Anzio Road, Observatory, 7925, Cape Town, South Africa; 2 Division of Clinical Pharmacology, Department of Medicine, University of Cape Town, 7925, Cape Town, South Africa

## Abstract

**Background:**

Within the context of increasing antimalarial costs and or decreasing malaria transmission, the importance of limiting antimalarial treatment to only those confirmed as having malaria parasites becomes paramount. This motivates for this assessment of the cost-effectiveness of routine use of rapid diagnostic tests (RDTs) as an integral part of deploying artemisinin-based combination therapies (ACTs).

**Methods:**

The costs and cost-effectiveness of using RDTs to limit the use of ACTs to those who actually have *Plasmodium falciparum *parasitaemia in two districts in southern Mozambique were assessed. To evaluate the potential impact of introducing definitive diagnosis using RDTs (costing $0.95), five scenarios were considered, assuming that the use of definitive diagnosis would find that between 25% and 75% of the clinically diagnosed malaria patients are confirmed to be parasitaemic. The base analysis compared two ACTs, artesunate plus sulfadoxine/pyrimethamine (AS+SP) costing $1.77 per adult treatment and artemether-lumefantrine (AL) costing $2.40 per adult treatment, as well as the option of restricting RDT use to only those older than six years. Sensitivity analyses considered lower cost ACTs and RDTs and different population age distributions.

**Results:**

Compared to treating patients on the basis of clinical diagnosis, the use of RDTs in all clinically diagnosed malaria cases results in cost savings only when 29% and 52% or less of all suspected malaria cases test positive for malaria and are treated with AS+SP and AL, respectively. These cut-off points increase to 41.5% (for AS+SP) and to 74% (for AL) when the use of RDTs is restricted to only those older than six years of age. When 25% of clinically diagnosed patients are RDT positive and treated using AL, there are cost savings per malaria positive patient treated of up to $2.12. When more than 29% of clinically diagnosed cases are malaria test positive, the incremental cost per malaria positive patient treated is less than US$ 1. When relatively less expensive ACTs are introduced (e.g. current WHO preferential price for AL of $1.44 per adult treatment), the RDT price to the healthcare provider should be $0.65 or lower for RDTs to be cost saving in populations with between 30 and 52% of clinically diagnosed malaria cases being malaria test positive.

**Conclusion:**

While the use of RDTs in all suspected cases has been shown to be cost-saving when parasite prevalence among clinically diagnosed malaria cases is low to moderate, findings show that targeting RDTs at the group older than six years and treating children less than six years on the basis of clinical diagnosis is even more cost-saving. In semi-immune populations, young children carry the highest risk of severe malaria and many healthcare providers would find it harder to deny antimalarials to those who test negative in this age group.

## Background

Malaria is a complex disease that varies in epidemiology and public health impact in different parts of the world. Estimates by the World Health Organization indicate that there are 350–500 million clinical cases of malaria each year [1]. These figures may be a significant under-estimate of the true malaria toll considering that the greatest impact of malaria occurs in areas where surveillance and reporting systems are weak. However, it is also possible that these statistics are over-estimated given that the areas where the greatest proportion of malaria cases occur rely mostly on clinical diagnosis of malaria, which usually includes many other acute febrile illnesses. Administration of antimalarial treatment has been predominantly based on clinical diagnosis [2-10]. More recently, however, the availability of rapid diagnostic tests (RDTs) has led to definitive diagnosis being considered as a strategy to target the use of antimalarials [3-12].

Clinical diagnosis has the key advantage of being cheap and easy to perform in rural settings and by those with limited training. In addition, the diagnosis is made relatively quickly (compared to definitive diagnosis where a health worker has to perform the test and/or wait for test results), allowing for quick provision of antimalarial treatment. Clinical diagnosis is, however, likely to result in erroneous treatment of millions of non-malaria cases. Misdiagnosis of malaria is costly and results in considerable morbidity and mortality, because it contributes to both a delay in treatment of the correct diagnosis and to increasing antimalarial drug pressure and thus resistance, thereby speeding up the obsolescence of affordable drugs [4,5].

Definitive diagnosis, when used correctly, can contribute to better and more cost-effective disease management and can reduce the unnecessary use of antimalarial drugs. Although microscopy is considered to be the gold standard for malaria diagnosis [2-5] and has several advantages over other diagnostic approaches, it has been found to be operationally impractical in rural or resource-poor settings due to its requirements for personnel, equipment, regular supply of reagents and continued quality assurance supervision [2-4]. Rapid diagnostic tests offer the possibility for accurate and accessible detection of malaria parasites, and have an important role in limiting malaria over-diagnosis and over-treatment [9-15], particularly where accurate microscopy is not accessible. Provided they do actually reduce the prescription of antimalarials, RDTs are expected to become more cost-effective as antimalarials become increasingly expensive and/or the risk of malaria is reduced. Extensive resistance to chloroquine, sulphadoxine-pyrimethamine (SP) and amodiaquine monotherapy has prompted malaria treatment policy change to more expensive combinations, especially artemisinin-based combination therapies (ACTs). Widespread use of artemisinin-based combination therapies has been shown to decrease malaria transmission in Zanzibar [16], South Africa [17] and Thailand [18]. Similarly, high coverage with effective indoor residual spraying programmes [19] or insecticide-treated bed nets [20,21] have resulted in sustained reductions in malaria risk.

The role of definitive diagnosis or rapid diagnostic tests is more uncertain in areas with high intensity malaria transmission. In these areas, it has been considered acceptable to treat on a clinical diagnosis on the grounds that a high proportion of febrile cases are actually parasitaemic and that it is better to treat all febrile cases than to miss one potentially fatal malaria infection, especially in young children [22]. On the other hand, it could also be argued that there is a greater likelihood for partial immunity in the adult populations living in areas of high transmission intensity and, therefore, it is likely that a smaller proportion of their fevers would actually be malaria cases.

Within the context of increasing antimalarial costs and/or decreasing malaria transmission, the importance of limiting antimalarial treatment to only those confirmed as having malaria parasites becomes paramount [2,4,9]. This provides the rationale for the assessment of the cost-effectiveness of routine use of RDTs as an integral part of deploying artemisinin-based combination therapies, particularly in context of low to moderate intensity malaria transmission.

## Methods

This study was undertaken in two districts in southern Mozambique (Namaacha and Matutuine). Both districts in Mozambique were in holoendemic malaria transmission areas with *Plasmodium falciparum *prevalence among children aged 2–15 years of over 60% prior to the implementation of a community based indoor residual spraying (IRS) programme [23]. The aim of this study was to determine whether RDTs should be implemented prior to the introduction of artesunate plus SP (AS-SP) to replace chloroquine as first-line malaria treatment and artemether-lumefantrine to replace SP as second-line treatment within the public sector. The costs and cost-effectiveness of using RDTs to limit the use of ACTs to those who actually have malaria, under different scenarios of malaria parasite prevalence (25% – 75%) among clinically suspected malaria cases were assessed. In addition, the analysis included an evaluation and comparison of two differently priced ACTs and assessed restricting RDTs to only those older than six years.

Before the implementation of ACTs and RDTs in the two pilot districts in southern Mozambique, relevant baseline data on malaria-related costs and health outcomes were collected from all the health facilities in the two districts, which comprised of 13 health posts (clinics) and two health centres (district hospitals). Costing was undertaken from a public sector provider's perspective and included the costs of RDTs and antimalarials for malaria outpatients. Capital costs were excluded from this analysis since RDTs do not require additional equipment or infrastructure.

The estimation of treatment costs for non-malaria febrile cases (those who are malaria test negative), some of whom would be treated for other illnesses, was beyond the scope of this study and is, therefore, not assessed or discussed further.

For this study, data on the number and age distribution of clinically diagnosed malaria cases for the year 2002 were obtained from the provincial and district Ministry of Health records in the 15 health facilities studied. Based on the age distribution observed from this facility data, the proportions of clinically diagnosed malaria patients in each age category were calculated for artesunate plus SP (Table 1). Proportions in the different age categories used for dosing artemether-lumefantrine were then extrapolated assuming even age distribution within each age category (Table 1).

**Table 1:** Age distribution of patients with clinically diagnosed malaria in Namaacha and Matutuine districts, southern Mozambique (n = 31,438)

**AS+SP**		**artemether-lumefantrine**		
**Age**	**Observed n (percentage)**	**Age**	**Estimated percentage**	Basis for calculation*

**1–6 years**	8,882 (28.3%)	**1–5 years**	23.6%	As for AS+SP, less 4.7% to cater for 6 year olds
**7–13 years**	4,814 (15.3%)	**6–8 years**	9.1%	As for AS+SP, plus 4.7% to include 6 year olds, minus 10.9% to exclude 9–13 year olds
**14+ years**	17,742 (56.4%)	**9–12 years**	8.7%	100% minus the other age categories' %
		**13+ years**	58.6%	As for AS+SP, plus 2.2% to include 13 year olds

	**100%**		**100%**	

* assuming even age distribution within each age category.

### Calculating costs

The calculation of costs is based on the number of suspected malaria cases (for the clinical diagnosis scenario) and on the calculated malaria cases for each scenario of definitive diagnosis considered (25% – 75% of suspected cases being confirmed malaria cases).

#### Cost of antimalarials

ACT dosages (and thus costs) are dependent on patient age (or weight) (see Table 1). Children under one year of age were excluded from this analysis as the Maputo provincial guidelines recommend that this high-risk group is admitted to the health centre for treatment with quinine.

The costs of AS+SP and artemether-lumefantrine have been calculated as *unit price *in 2004 (see Table 2) multiplied by the *estimated quantity of antimalarials consumed*. The unit price of artesunate is based on the cost of $0.10 per 50 mg tablet for the only artesunate product pre-accredited by the WHO at the time of the study (Arsumax^®^, Sanofi-Aventis, Paris, France). The price at which Mozambique ordered SP (Fansidar^®^, a fixed dose combination of 500 mg sulphadoxine plus 25 mg pyrimethamine manufactured by Roche, Johannesburg South Africa) for the pilot ACT deployment of $0.19 per tablet, was used in the analyses. The unit prices of artemether-lumefantrine (Coartem^® ^20 mg artemether and 120 mg lumefantrine, Novartis, Basel, Switzerland) are based on the WHO preferential price at the time of the study of $2.40 per adult treatment [24]. Sensitivity analyses included the subsequently reduced WHO preferential prices of AL of $1.44 per adult treatment. *Quantities of antimalarials consumed *were calculated with the assumptions that malaria patients would receive the recommended doses of antimalarials and that there was no wastage of antimalarials.

**Table 2:** Unit prices for antimalarials

**Age Group**	**Price per treatment course (AS+SP)**	**Number of tablets for full treatment course**	**Age (Weight) Group**	**Price per treatment course (AL)**	**Number of AL tablets for full treatment course**
1–6 years	$0.49	3 AS + 1 SP	10–14 kg (1–5 years)	$0.90	6
7–13 years	$0.98	6 AS + 2 SP	15–24 kg (6–8 years)	$1.40	12
14+ years	$1.77	12 AS + 3 SP	25–34 kg (9–12 years)	$1.90	18
			35+kg (13+ years)	$2.40	24

#### Cost of RDTs

The cost of RDTs was calculated as the *unit price of RDTs *multiplied by the *estimated quantity of RDTs used*. For previous surveys in the study area, the Mozambican Ministry of Health was using RDTs (ICT Diagnostics PF Tests ML01^®^) for which a box of 25 RDT tests was obtained at a price of $23.72. This translates into a unit price of USD 0.95 (2003 prices). *Quantity of RDTs used *was estimated assuming that each clinically diagnosed malaria case would be tested using one RDT.

#### Costing under the different scenarios

Given the reliance on clinical diagnosis of malaria, actual malaria incidence in most sub-Saharan countries is not known. Analyses presented in this paper focus on a wide range of scenarios reflecting varying levels of malaria endemicity and consider that between 25% and 75% of the clinically diagnosed cases would be malaria positive when an RDT is used. It was assumed that these proportions were constant across all age groups, although in areas of intense malaria transmission the proportion of RDT positive patients may be expected to decrease with age as partial immunity is required. To address this, the study included an analysis of the costs and cost-effectiveness of limiting RDT use to patients over six years of age. The cost-effectiveness of RDTs is largely driven by the cost of treatment (ACTs) relative to the cost of diagnosis. The rationale for using the cut-off as six years of age was because of the age-based dosage for artesunate plus SP, the ACT planned for deployment in Mozambique, increasing from one tablet ($0.49) for children aged 1 – 6 years to two tablets ($0.98) for children aged 7 – 13 years. Since the cost of RDTs ($0.95) remains constant regardless of age, the possibility of RDTs being cost saving would only arise for children over six years of age when artesunate plus SP is treatment policy.

**Total cost savings (or incremental costs) **were calculated as the difference between the total costs of RDTs and antimalarials under the different RDT scenarios and total antimalarial costs if clinical diagnosis was used. The incremental costs per malaria positive case treated (ICER) has been calculated as incremental costs divided by number of malaria positive cases treated for a given scenario.

### Sensitivity analyses

The initial set of assumptions, as described in the methodology above, is referred to as the 'base case' in the results section. One-way and multi-way sensitivity analyses were performed to assess whether the results are sensitive to changes in:

• The price of RDTs: the lowest 2004 cost of quality-assured rapid diagnostic tests, which was US$ 0.65 (e.g. Paracheck^®^, Orchid Biomedical, India) was used;

• The price of ACTs: Prices of ACTs lower than the ones in the base case scenario have been considered for sensitivity analyses. The preferential price of artemether-lumefantrine to the WHO has been reduced to $1.40 per adult treatment course plus a 3% fee levied by WHO to cover shipping costs. This provided the basis for the sensitivity analyses using $1.44 per adult treatment. The international median price per tablet of SP USD 0.0257 [25] and of artesunate costing $0.077 per tablet were also considered; and

• The age distribution of febrile patients: Three alternative age distributions were explored in the sensitivity analysis (see Table 3), increasing the proportion of children in Age Breakdown 1 and 2, and increasing the adult population in Age Breakdown 3. These reflect populations with higher birth rates and those dominated by adult migrant workers, respectively.

**Table 3:** Age breakdown for base case and for three sensitivity analyses

	Artesunate plus SP			Artemether – lumefantrine			
Age (years)	1 – 6	7 – 13	14+	1 – 5	6 – 8	9 – 12	13+

**Base case**	28.3%	15.3%	56.4%	23.6%	9.1	8.7	58.6
Age breakdown 1	50%	15%	35%	45%	9%	9%	37%
Age breakdown 2	40%	20%	40%	35%	11.5%	11.5%	42%
Age breakdown 3	10%	5%	85%	8%	2%	2%	88%

The values used in the multi-way sensitivity analyses for each variable are summarised in Table 4.

**Table 4:** Values of the variables considered for the sensitivity analyses

	**Price of SP (per tablet)**	**Price of AS (per tablet)**	**Price of AL (per adult dose)**	**Price of RDTs**	**Age breakdown**
**Base case**	$ 0.19	$ 0.10	$ 2.40	$ 0.95	base case
**Multi-way 1**	$ 0.0257	$ 0.077	$ 1.44	$ 0.65	1
**Multi-way 2**	$ 0.19	$ 0.10	$ 2.40	$ 0.95	3
**Multi-way 3**	$ 0.19	$ 0.10	$ 2.40	$ 0.65	3
**Multi-way 4**	$ 0.0257	$ 0.077	$ 1.44	$ 0.65	base case

## Results

### Total costs and cost savings associated with the use of RDTs

**Figure 1:**
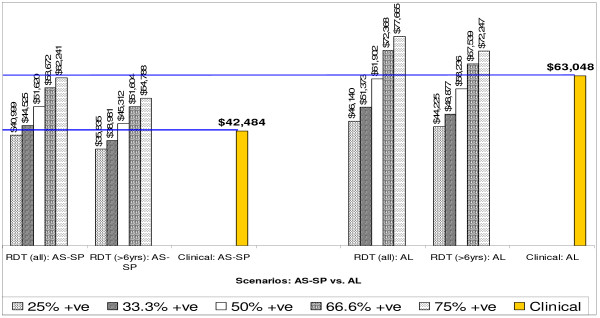
Total cost of antimalarials and RDTs: comparing clinical and definitive diagnosis.

Figure 1 shows that the total costs of antimalarials and RDTs for treating all malaria patients in 2002 in the two study districts. Using clinical diagnosis, this would be $42,484 when treating them with AS+SP and $63,048 for AL. The introduction of definitive diagnosis (using RDTs) could either result in cost savings or additional costs, depending on the proportion of febrile patients confirmed to have malaria. For the scenario where 25% of febrile cases are RDT positive, use of definitive diagnosis before treating patients would result in a cost saving of up to $1,485 and $16,908, when malaria patients are treated with AS+SP and AL, respectively, provided that health workers do not give antimalarials to patients with a negative RDT. Thus the more expensive the antimalarial being used, the greater the need for restricting antimalarials to confirmed malaria cases and the higher the cost savings that will be realised through effective implementation of definitive diagnosis.

### Incremental costs or cost savings associated with the use of RDTs

**Figure 2:**
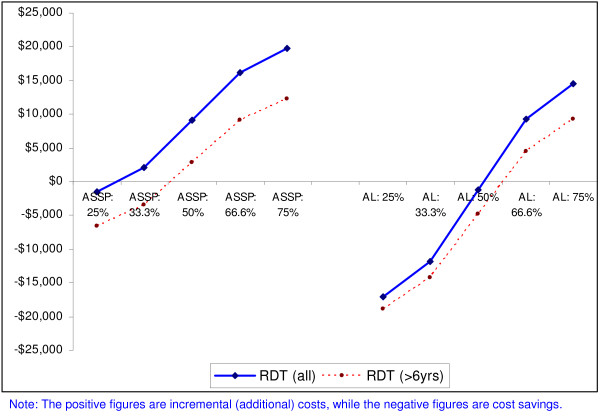
Incremental costs (or cost savings) associated with use of RDTs when malaria is confirmed in varying proportions of patients for two ACTs (artesunate plus sulfadoxine-pyrimethamine and artemether-lumefantrine); excluding 'other recurrent costs'.

Figure 2 shows the incremental costs (or cost savings) for antimalarials and diagnosis under the different scenarios. For the relatively cheaper ACT (AS+SP), only when 29% or less of all suspected malaria cases test positive for malaria will the use of RDTs in all clinically diagnosed malaria cases result in cost savings, when compared to all patients being treated with AS+SP on the basis of clinical diagnosis. This percentage increases from 29% to 41.5% when use of RDTs is restricted to only those older than six years of age, and malaria positive patients are treated with AS+SP. For a relatively more expensive ACT (e.g. artemether-lumefantrine in 2004), as long as fewer than 52% of tested cases are found to be positive, the use of RDTs in all suspected malaria cases will result in lower treatment costs (cost savings) compared to when patients are treated on the basis of clinical diagnosis; this cut-off shifts from 52% to 74% if use of RDTs is limited to patients who are over six years of age. This strategy results in lower additional costs or higher cost savings compared to when RDTs are used in all suspected malaria cases, for both AS+SP and artemether-lumefantrine. However, in terms of cost, there are greater gains in restricting use of RDTs in patients over six years of age, when treating with a less expensive ACT (e.g. AS+SP). This is expected since the price of one RDT ($0.95) is nearly twice as high as the cost of one dose of AS+SP for a patient younger than or equal to six years ($0.49), but similar to the cost of an AL treatment course for this age group ($0.90). Hence, treating all patients younger than or equal to six years with AS+SP on a clinical basis makes more economic sense than using an expensive RDT to test this age group.

### Cost-effectiveness of RDTs: incremental cost of per malaria patient treated

**Figure 3:**
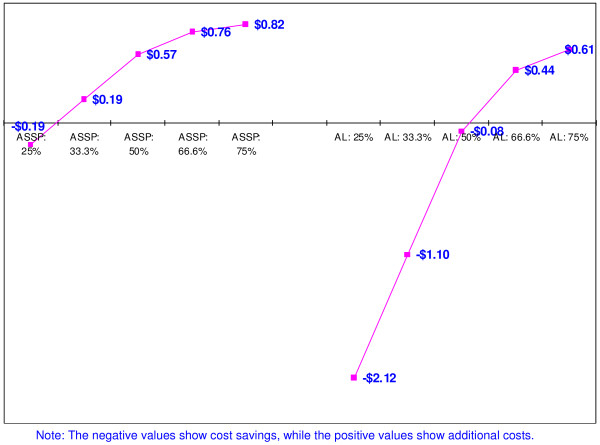
Incremental costs of using RDTs per malaria positive patient treated (base case): based on total costs of RDT and antimalarials (artesunate plus sulfadoxine-pyrimethamine and artemether-lumefantrine) when all suspected malaria cases are tested.

Figure 3 presents results on the incremental costs *per malaria positive patient treated*. Incremental costs have been calculated using total cost of RDTs and antimalarials divided by the number of malaria cases for the different scenarios. Findings reported are based on the assumption that health workers adhere to test results and do not give antimalarials to patients with a negative RDT. Results in Figure 3 again show a cost saving (of $0.19 per patient treated) if malaria is present in under 29% of patients and that even when 75% of cases are malaria positive, the *incremental cost per malaria positive patient treated *is less than US$ 1, when AS+SP is used for treating malaria patients.

When patients are treated using artemether-lumefantrine, there are *cost savings per malaria positive patient treated *of up to $2.12 (in the 25% scenario) as long as 52%, or less, of the suspected cases are RDT test positive. Beyond the 52% cut-off point, additional costs are incurred with an *incremental cost per malaria positive patient treated *of up to $0.85 (when 95% of tested cases are found positive and treated with artemether-lumefantrine). According to the guideline provided by the Ad Hoc Committee on Health Research relating to Future Interventions Options, an intervention is considered to be "*highly attractive*" (hence 'cost-effective') in low income countries if it costs less than $25 per disability-adjusted life year (DALY) averted and any intervention that costs less than $150 per DALY averted should be considered "attractive" [26]. Although the health outcome used in this analysis is "number of patients treated" and not DALYs, this guideline could be helpful in considering whether an *incremental cost per malaria positive person treated *of less than $1 should be regarded as being highly cost-effective.

### Sensitivity analyses

**Figure 4:**
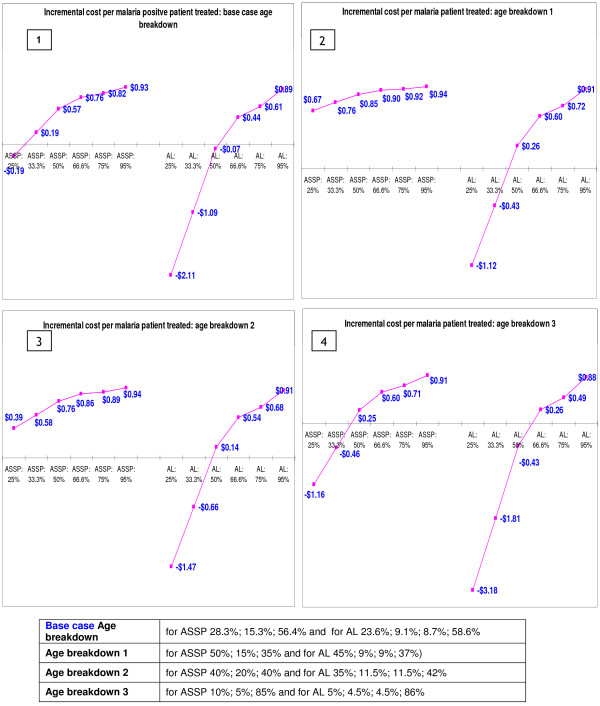
Impact of changes in age distribution of suspected malaria cases on incremental cost per malaria patient treated.

**Figure 5:**
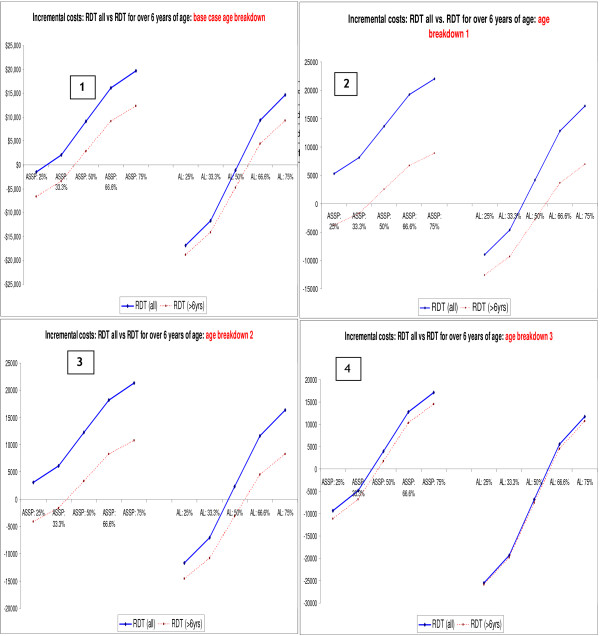
Impact of changes in age distribution of suspected malaria cases on incremental costs of RDTs + antimalarials (excluding other recurrent costs).

Findings from the one-way sensitivity analysis on variation in the *age distribution *show that the higher the percentage of adults among the suspected malaria cases (age breakdown 3), the lower the additional costs and the higher the costs savings (particularly with a relatively more expensive ACT like AL) associated with use of RDTs (Figure 4, quadrant 4), and vice versa. This finding is not surprising since the price of the ACTs for children is significantly lower than the price of the adult dose, and yet the price of the RDT remains constant for all age groups. Results in Figure 5 show how changes in the *age distribution of patients *with clinically suspected malaria have an impact on the decision on restricting their use to only those who are over six years of age. The higher the proportion of young children among those with suspected malaria, the more it makes economic sense to restrict the use of RDTs to those over the age of six years. The more expensive the unit price of the antimalarial for the one to six years age group, relative to the unit price of RDTs, the lower the cost savings associated with restricting RDTs to patients over six years of age (Figure 5).

**Figure 6:**
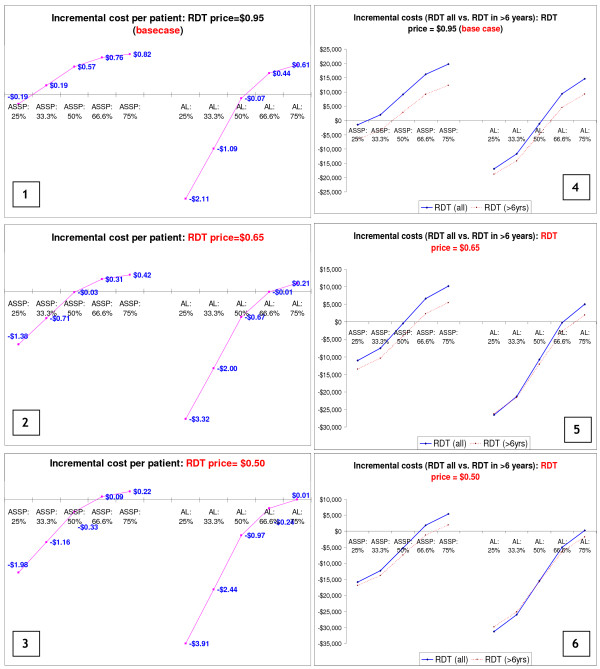
Impact of changes in price of RDTs on Incremental cost per patient treated and total incremental costs.

Results of the one-way sensitivity analysis on the *RDT price *variable show that as expected, the lower the unit price of a rapid diagnostic test the more cost-effective it is to use definitive diagnosis (using RDTs) as the basis for ACT treatment, regardless of the price of the antimalarial being used. With a reduction in the unit price of RDTs from $0.95 to $0.50, limiting the use of RDTs in patients older than six years would result in significantly less economic gains (when patients are treated with AS+SP) and some economic losses in areas of low to moderate intensity malaria transmission (where 50% or less of fever cases are malaria positive). The same applies when patients are treated with AL (quadrant 6, Figure 6). In other words, the cheaper the RDT the less the need is to restrict to older age groups.

Similarly, results of the one-way sensitivity analysis on the *ACT price *variable show, as expected, that the use of RDTs will become less cost-effective as the antimalarials become less expensive. This explains why, at least from an economic perspective, RDTs have not been widely used when cheaper antimalarials, such as chloroquine or sulfadoxine-pyrimethamine monotherapy, were being used in areas of moderate to high intensity malaria transmission. This may also be the "in-country" scenario with the implementation of a global subsidy to reduce the price of ACTs to that of chloroquine [27].

**Figure 7:**
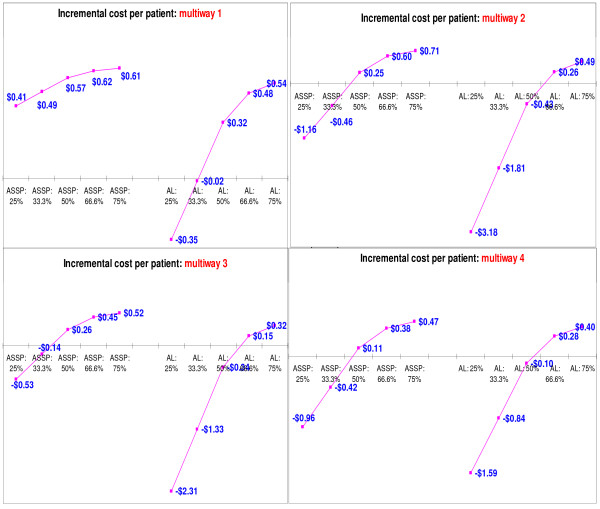
Multi-way sensitivity analyses: Incremental cost per malaria patient treated.

Results of the multi-way sensitivity analyses (Table 4) are presented in Figure 7. In these analyses the prices of antimalarials, prices of RDTs and age distribution were varied to assess the effect of simultaneous changes in these variables on the earlier findings on cost-effectiveness of RDTs. Figure 7 shows that, as expected, *multi-way 1 *(high prices of ACTs and RDTs and children younger than or equal to six years of age taking up the highest proportion) is the context in which routine use of RDTs is least cost saving (for both AS+SP and artemether-lumefantrine) (quadrant 1, Figure 7). Results of *multi-way 2 *sensitivity analysis show the impact of changing age distribution alone (without changing the prices of ACTs and RDTs). There is a decline in incremental cost per patient treated, from $0.82 to $0.71 and from $0.61 to $0.49 for AS+SP and artemether-lumefantrine respectively, purely as a result of increasing the proportion of adults in the population with clinically diagnosed malaria.

Results of multi-way 3 sensitivity analysis (quadrant 3, Figure 7) show the impact of changing the price of RDTs and age distribution (without changing prices of ACTs). Changes in costs are mainly due to the variations in RDT prices and the proportions of adults treated. As expected, the bigger the proportion of the suspected cases that are adults, the greater the cost savings. Variation in the prices of RDTs and antimalarials shifts the cut-off points at which definitive diagnosis results in cost savings.

## Discussion

With improvements in malaria control and relatively higher costs of antimalarial treatment, there is increased opportunity for cost savings through the introduction of rapid diagnostic tests in facilities and/or communities where microscopic confirmation of malaria diagnosis is not reliably available [28]. Amexo and others suggest that it is unethical to continue with high levels of malaria misdiagnosis in light of the introduction of expensive antimalarials and availability of cost-effective methods of diagnosing malaria (such as RDTs) [7]. This argument in favour of limiting antimalarial use to confirmed cases is strengthened by considering the enormous effect of drug pressure on antimalarial resistance and the potential for adverse reactions. This study shows that the introduction of RDTs is likely to be cost saving when a relatively more expensive ACT is used for treatment, provided no more than 52% of those patients clinically diagnosed to have malaria are found to be parasitaemic. This result holds even when relatively less expensive ACTs (e.g. AS+SP or the current preferential price to WHO for AL) are used, but the higher the price of the ACT, the greater the cost savings from introducing definitive diagnosis and the higher the cut-off point at which RDTs become cost-saving. Many countries in Africa have well below 60% of clinically diagnosed malaria patients being confirmed on RDT or microscopy. A study in Uganda found that only 57% of those clinically diagnosed as having malaria were actually parasitaemic [29]. From several studies undertaken separately in 15 countries, Amexo and others (2004) report that on average there is a 61% overestimation of malaria cases when clinical diagnosis is used [7].

As malaria control improves as a consequence of widespread use of insecticide treated bednets [30], indoor residual spraying [23] and or ACT use [16-18,31], the proportion of clinical malaria (fever) cases that would be definitively diagnosed as malaria will decrease. Consequently the introduction of definitive diagnosis with RDTs at the same time as the introduction of ACT would become increasingly cost saving, because there would be fewer suspected cases to test and even fewer cases to treat. Findings from our study show that the introduction of definitive diagnosis (using RDTs) is cost-saving even when we only consider a narrow perspective of costs related to malaria treatment (i.e. costs of RDTs and antimalarials).

While the use of RDTs in all suspected cases has been shown to be cost-saving in some instances, our findings also show that targeting RDTs at the group older than six years and treating all children less than six years on the basis of clinical diagnosis is even more cost-saving. In semi-immune populations, young children carry the largest malaria disease burden and many healthcare providers would find it harder to deny antimalarials to those who test negative in this age-group.

Findings from the analyses show that results are sensitive to the effects of changes in *age distribution of the symptomatic population*, and *price of ACTs *and *price of RDTs*. Increasing proportions of older patients result in increased cost savings with RDT introduction (due to lower costs of antimalarials for children, and fixed cost of RDTs). As the prices of antimalarial treatment increase, RDT implementation becomes increasingly cost saving. However, when less expensive ACTs are introduced, such as for the current WHO preferential price of $1.44 per adult treatment, the RDT price to the healthcare provider should be $0.65 or lower for RDTs to be clearly cost saving in populations with between 30% and 52% of clinically diagnosed malaria cases being confirmed malaria cases.

The analysis of the potential benefits of introducing RDTs in combination with ACTs is more complex if the majority of malaria treatment is self-administered or sought in the private and informal sectors. Furthermore, for treatment of fever cases testing negative for malaria, some healthcare providers may still use antimalarials and there may be an increased risk of irrational use of other drugs, particularly antibiotics. This necessitates integrated training and supervision of health workers in rational drug use for the treatment of non-malaria fever cases and enhanced drug utilisation monitoring, particularly at the time of RDT introduction. Village health volunteers in Laos were found to only require minimal training (one hour) to sustain reliable use of RDTs over a 10 month period [32]. In contrast, a study conducted in Zambia, where specific training and supervision of healthcare providers was not provided, significant underutilization of RDTs or the inappropriate prescription of antimalarials when patients test negative have been documented [5]. However, a study in Tanzania concluded that use of rapid diagnostic tests, with a single training emphasising that "negative malaria tests should lead to alternative diagnoses being considered", did not lead to lead to any reduction in over treatment for malaria [6].

Although the additional benefits of avoiding the use of ACTs to patients in whom the malaria diagnosis is negative could not be included in this study, these are likely to be substantial. Assuming education interventions succeed in ensuring that test results are accepted by healthcare providers, patients and caregivers, excluding malaria would facilitate earlier diagnosis and treatment of the actual cause of the disease for those who are malaria test negative, and would minimise the treatment seeking costs related with repeat visits and the productivity losses associated with prolonged illness. It has been argued that the effects of malaria misdiagnosis fall most heavily on the poor and vulnerable who are least able to withstand prolonged ill-health and the associated missed opportunities for earning an income [9]. In addition, the use of definitive diagnosis would provide more reliable data on malaria cases, hence allowing for accurate forecasting of required antimalarials for planning and budgeting and better monitoring of the effectiveness of malaria control interventions. Also, drug pressure, and consequently, the rate of spread of antimalarial resistance could be decreased.

The findings of this study show that the introduction of RDTs is likely to be cost saving when ACTs are implemented, particularly in areas of low to moderate intensity malaria transmission. The finding that drug cost-savings are higher in low transmission areas was also reported by Goodman and others [1]. This is because in low transmission areas, a higher proportion of malaria cases are adults who require a higher antimalarial dose (and for whom antimalarials thus cost more). This effect results in higher malaria prevalence cut-off point at which RDTs are cost effective. Similar findings are reported by Rolland and others where the cost-effectiveness of RDTs is studied in the context of malaria epidemics. They report that RDTs would be cost-effective at a malaria prevalence of up to 45% when AS+AQ is used for treatment and up to 68% when artemether-lumefantrine is used [33].

It is also important to note that clinical diagnosis may fail to pick up some patients who actually have malaria. Luxemburger and others found that none of the malaria symptoms alone or in combination proved to be a reliable predictor of malaria, and at best clinical diagnosis would result in prescription of antimalarials in 29% of the non-malaria febrile illnesses and 49% of the true malaria cases (suggesting that 51% of the infections would go untreated initially) [7]. It is important to note that RDTs are unlikely to be used if malaria is not clinically suspected and so will not address the problem of false negatives arising from clinical diagnosis. Furthermore, the extent to which RDTs are cost-effective depends on their accuracy in diagnosing malaria. Studies have found varying levels of sensitivity and specificity of the different RDT products on the market. Swarthout and others (2007) recently reported 52% specificity of Paracheck-Pf in Democratic Republic of Congo and noted that as many as 92% of children were still false positive at day 28 following treatment with artemether-lumefantrine [34]. Similar results are reported by Kleinschmidt and others (2007) for Equatorial Guinea [35].

## Conclusion

Rapid, accurate and accessible detection of malaria parasites has an important role in addressing the problem of malaria over-diagnosis and inappropriate use of antimalarial drugs. RDTs offer the potential to provide accurate diagnosis to all populations at risk, particularly those unable to access good quality microscopy services. In the context of expensive antimalarial drugs (such as ACTs), deploying RDTs can be cost-saving or cost-effective depending on the price of RDTs and ACTs, the age distribution of and the prevalence of malaria parasites among clinically diagnosed malaria patients. This result holds true only if health workers prescribe and or dispense antimalarials to only the patients that are found to be malaria test positive.

However, in most malaria endemic countries, access to preventive, diagnostic and curative services remains limited due to a range of access constraints, including health service costs. Care should be taken to ensure that the costs of new and apparently cost-saving interventions, such as RDTs, are not borne by households. In this era where the use of ACTs is being globally encouraged and financed, strategies such as the concurrent use of ACTs and RDTs should be equally encouraged and financially supported unless the majority of clinically diagnosed malaria cases are parasitaemic.

## Competing interests

The authors declare that they have no competing interests.

## Authors' contributions

All authors had equal contribution in preparing this article. CMZ undertook all the data collection, entry and analysis for this study, and received continuous technical support from DM, who was integrally involved in the conceptualization of the analytic approach. CMZ also drafted the first manuscript of this article based on her PhD thesis, which was supervised by DM and KB. KB contributed to the study design and made substantial edits to the first draft of this article. KB and DM have both been involved in reviewing subsequent drafts of this article and have provided on-going guidance to CMZ. All authors have contributed to finalizing the article.
